# Profiling and Bioinformatic Analyses Indicate Differential circRNA and miRNA/isomiR Expression and Interactions

**DOI:** 10.1155/2018/8518563

**Published:** 2018-02-25

**Authors:** Li Guo, Liang Zheng, Yang Zhao, Qianyun Wang

**Affiliations:** ^1^Department of Bioinformatics, School of Geographic and Biologic Information, Nanjing University of Posts and Telecommunications, Nanjing 210023, China; ^2^Department of Cardiothoracic Surgery, The Third Affiliated Hospital of Soochow University, Changzhou 213003, China; ^3^Department of Biostatistics, School of Public Health, Nanjing Medical University, Nanjing 211166, China

## Abstract

As a novel class of noncoding RNAs, circular RNAs (circRNAs) have been reported to play a role in various biological processes. Some circRNAs may serve as microRNA (miRNA) sponges, regulating transcription or splicing. Herein, we investigated the expression profiles and interactions of miRNAs/isomiRs and circRNAs in male patients with esophageal cancer. We found that some miRNA genes generated two deregulated miRNA products (miR-#-5p and miR-#-3p), and these products were consistently abnormally expressed. Some circRNAs were predicted to be miRNA sponges for specific miRNAs. Some of these typically showed opposing expression patterns in cancer tissues: one upregulated and the other downregulated. Although fewer miRNAs were predicted to interact with circRNAs, the number of predicted interactions would be substantially increased if detailed isomiRs were involved. High sequence similarity across multiple isomiRs suggested that they might interact with circRNAs, similar to the interaction of homologous miRNAs with circRNAs. At the isomiR level, due to the characteristics of the sequences and expression patterns involved, the cross-talk between different ncRNAs is complicated despite simplification of the isomiRs involved through clustering. We expect that our results may provide methods for further study of the cross-talk among ncRNAs and elucidate their biological roles in human diseases.

## 1. Introduction

Esophageal cancer is a common cancer with high cancer mortality [1]. The impact of this disease is most severe in southern Africa, eastern Africa, and eastern Asia [2]. In China, the incidence of esophageal cancer in some areas is high [3]. Noncoding RNAs (ncRNAs), including microRNAs (miRNAs), long noncoding RNAs (lncRNAs), and circular RNAs (circRNAs), may play crucial roles in the regulation of the occurrence and development of many cancers. Indeed, some ncRNAs have been proposed as biomarkers for the diagnosis of many cancers [4, 5].

Among the ncRNAs, miRNAs have been shown to play crucial regulatory roles in a variety of biological processes, particularly those related to pathological and physiological pathways [6, 7]. An increasing number of studies have shown that miRNA loci can generate miRNA variants (isomiRs) [8–15]. It has further been shown that these isomiRs are functional molecules that have expression and sequence heterogeneities [12, 16–25]. Some small RNAs have been shown to have important regulatory roles in occurrence and development of esophageal cancer. For example, miR-330-3p regulates PDCD4 and might have an oncogenic role in esophageal squamous cell carcinoma [26], circuit of miR-31, SOX4, and EZH2 increases tumor progression in invasive esophageal carcinomas [27], and miR-10b may target KLF4 and thus promote the migration and invasion of tumorous cells [28]. These findings suggest that the small RNAs play important roles in esophageal cancer. However, few studies have investigated isomiRs, despite the fact that the canonical miRNA sequence is only one specific variant of multiple possible isomiRs [14, 29].

Another class of recently discovered animal RNAs, circRNAs, forms covalently closed continuous loops lacking 5′ and 3′ ends; these are also potential regulatory molecules [30]. Similar to miRNAs, circRNAs are thought to be crucial to cancer development and have thus been widely studied: a series of dysregulated circRNAs have been identified as important to the development of radiation resistance [31], circ_002059 is significantly downregulated in gastric carcinoma and may be a stable and novel biomarker [32], and circRNA_001569 may target miR-145 and thus contribute to colorectal cancer cell proliferation and invasion [33].

An understanding of miRNA-circRNA interactions is important for understanding the roles of ncRNAs in general, especially with respect to cross-talk among ncRNAs. Some circRNAs have been shown to have important regulatory roles in miRNA activity via sponging miRNAs. For example, the circRNA CDR1 acts as a miR-7 super-sponge [30, 34–36]. Similar to miRNAs, the expression profiles of circRNAs are specific to different cell types and tissues [30, 37], indicating potential regulatory functions and roles in cellular processes. Some studies have suggested that circRNAs may be potential biomarkers for disease diagnosis [32, 38], based on their potential biological roles and on their stable features, such as closed RNA loops.

Although miRNAs and circRNAs have been studied in many cancers, few have been investigated at the isomiR level, despite the fact that multiple isomiRs are the detailed products from miRNA locus. Herein, we aim to understand the expression profiles of miRNA/isomiR and circRNA in esophageal cancer using deep sequencing data, focusing on their interactions and potential roles in cancer development. We also propose an approach for the analysis of miRNA/isomiR-circRNA cross-talk.

## 2. Materials and Methods

### 2.1. Patient Samples

A total of 5 paired tissue samples (tumor tissue and matched adjacent nontumor normal tissue) were taken from 5 male patients (age: 64.2 ± 2.95) with squamous esophageal cancer (in all cases, the distance between the tumor and nontumor normal tissue was >5 cm, Table S1). The 5 patients underwent surgical resection without prior radiotherapy or chemotherapy, in the Department of Cardiothoracic Surgery at the Third Affiliated Hospital of Soochow University, Changzhou, China. All samples from patients were HPV-negative and HPV has been suggested to play an important role in the development of esophageal cancer [39]. Informed consent was obtained from each subject. This study was approved by the ethics committee of Soochow University.

### 2.2. High-Throughput Sequencing and Analysis

Total RNA was extracted from each sample using Trizol (Invitrogen, USA). Purified RNA was quantitatively and qualitatively analyzed with NanoDrop (Thermo Company, USA). CircRNA and small RNA sequencing libraries were constructed with HiSeq 2000 sequencing platform following the manufacturer's instructions.

CIRCexplorer software (version 1.1.7) [40] was used to identify circRNAs based on the fusion gene. After trimming adaptors, the small sequence fragments were mapped onto the human genome using Bowtie [41]. miRNAs/isomiRs were identified based on the canonical miRNA and pre-miRNA sequences annotated in miRBase version 21.0 (http://www.mirbase.org/) [42]. IsomiRs were identified according to the location of the canonical miRNA in pre-miRNA with ±4 nt [43], and further analyses were performed from the miRNA and isomiR levels, respectively. Differentially expressed circRNA and miRNA/isomiR profiles were obtained with edgeR, particularly those that were highly expressed. In the small RNA analysis, the canonical miRNA sequence and isomiRs from the specific miRNA locus were analyzed, respectively.

### 2.3. miRNA/isomiR-circRNA Interaction Analysis

**Figure 1:**
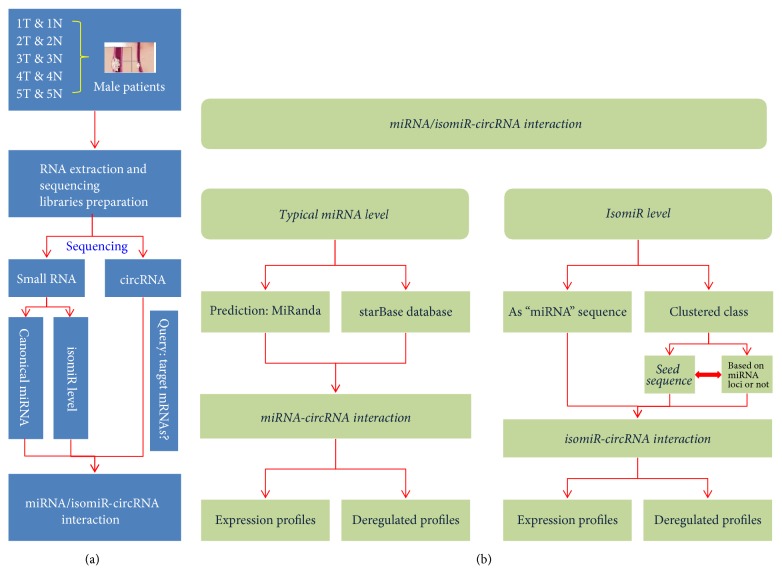
Flowchart for miRNA/isomiR-circRNA interaction. (a) Flowchart. Cancer and adjacent-normal samples were obtained from 5 male patients, and small RNA expression profiles and circRNA expression profiles were obtained via sequencing. (b) The detailed integrative analysis of small RNAs and circRNAs from the miRNA and isomiR levels.

The potential binding sites of abnormally expressed miRNAs were predicted using MiRanda [44] based on deregulated circRNAs. The potential relationships of different RNAs were constructed using Cytoscape version 3.4.0 [45]. It is possible to predict specific interactions at the isomiR levels, as well as based on the canonical miRNA sequence, or via clustering analysis of multiple isomiRs based on their close seed sequences (close sequence relationships can be obtained based on miRNA locus or independent miRNA locus) (Figure 1). Potential miRNA-circRNA interactions were also predicted and obtained from starBase v2.0 [46].

### 2.4. Functional Analysis of Differentially Expressed miRNAs and isomiRs

For the top differentially expressed miRNAs (the canonical miRNA and isomiRs), we performed functional analysis using CapitalBio Molecule Annotation System V4.0 (MAS, http://bioinfo.capitalbio.com/mas3/). Target mRNAs of the miRNAs were either identified from experimentally validated annotated in miRTarBase [47] or predicted with TargetScan [48]. Based on the same predictive methods, the target mRNAs of the isomiRs were predicted after clustering multiple isomiRs using seed sequences. We also further discussed the potential relationships among miRNAs/isomiRs, mRNAs, and circRNAs based on their functional relationships.

### 2.5. Statistical Analysis

Differentially expressed circRNAs and miRNA/isomiRs were obtained using edgeR based on paired* t*-test results with FDR correction: that is, when *P*_adj_ value < 0.05 and the |log_2_⁡(FC) | ≥2.0, we considered the RNA a deregulated species. Relative expressions were described using mean ± SD ($\overset{-}{x}\pm SD$) based on normalized sequence counts. Specifically, the relative expression of the isomiR was further estimated based on percentage of multiple isomiRs in the specific miRNA locus, and those abundantly expressed isomiR species were specifically concerned in relevant analysis. Distributions of different small RNAs were estimated using Venny [49].

## 3. Results

### 3.1. Overview of circRNA and miRNA/isomiR Expression Profiles Showed Abundant ncRNA Expression

Although some circRNAs were detected in both of the paired tumor and normal samples, some were expressed differentially in the tumor samples as compared to the normal samples based on the detected circRNAs from sequencing data (Figures S1 and S2). Among the small ncRNAs with abundant detection, many miRNA/isomiRs showed significant abnormal expression (Figure S3), and the isoform profiles were consistent with the relative expression levels of the miRNA locus.

We identified several pre-miRNAs capable of generating two mature miRNAs from both 5p and 3p arms. Interestingly, in deregulated miRNAs, based on canonical miRNAs, we found that some pairs of miR-#-5p and miR-#-3p showed consistent downregulation, including miR-204-5p (log_2_⁡FC = −3.41, FDR < 0.0001) and miR-204-3p (log_2_⁡FC = −3.17, FDR = 0.0146). This pattern was not detected in upregulated miRNA species.

### 3.2. Deregulated Small RNAs Contributed to Basic Biological Progress Based on Functional Analysis

**Figure 2:**
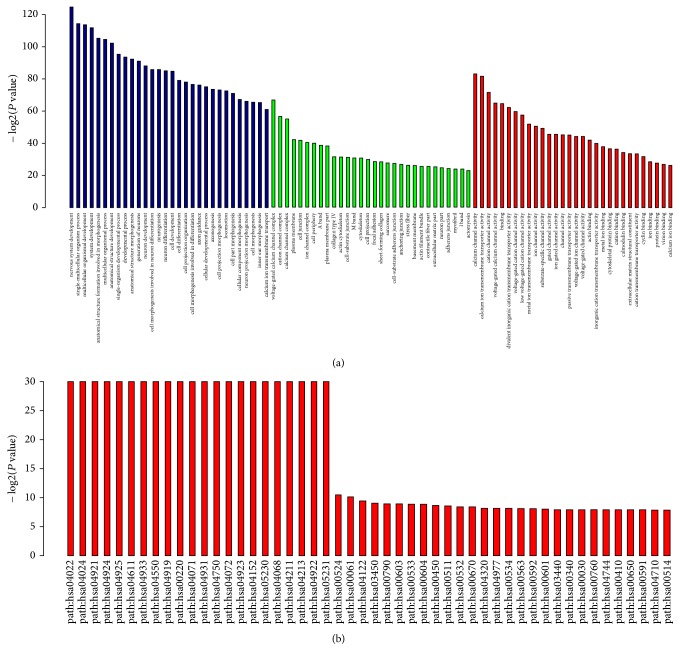
Functional enrichment analysis based on deregulated miRNAs. (a) Enriched pathways based on GO (blue column: some of the top biological processes; green column: some of the top cellular components; red column: some of the top molecular functions). (b) Enriched pathways based on KEGG.

Further functional analysis suggested that deregulated small RNAs might contribute to some basic biological progress via the negative regulation of target mRNAs (Figure 2). For example, these small RNAs might be involved in processes such as single-multicellular organism process, system development, cell development, and cell differentiation. Although the role of miRNAs in these pathways was indirect, their flexible regulatory contributions might be important to many relevant biological processes.

### 3.3. Predicted miRNA-circRNA Interaction Based on the Canonical miRNA

**Figure 3:**
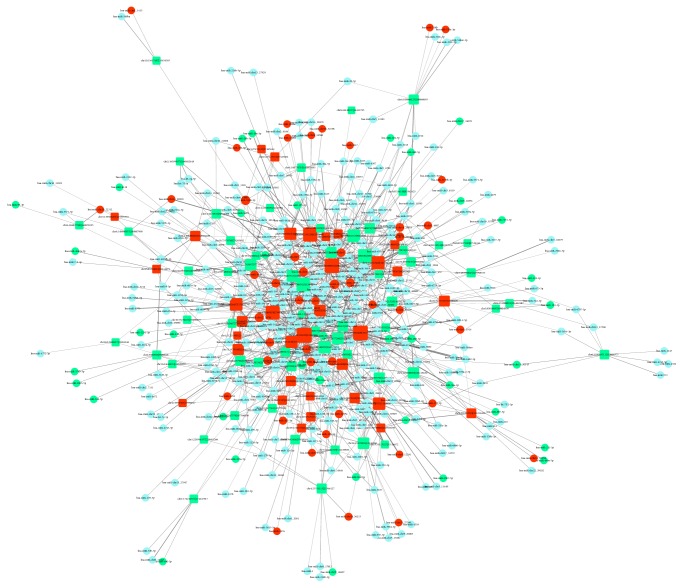
Predicted miRNA-circRNA interaction network. Square indicates circRNA, and circle indicates miRNA. Red indicates significantly upregulated RNAs, green indicates significantly downregulated RNAs, and light blue indicates no significant change in expression.

Based on known mature miRNAs and predicted circRNAs, we identified miRNA-circRNA pairs that potentially interact. Of these, some miRNAs were homologous with high sequence similarity (some nucleotides might vary, but these varied nucleotides were not located in the functional seed sequence). Homologous miRNAs might have been generated by homologous miRNA genes or by multiple pre-miRNAs. A total of 93 predicted circRNAs and 1,399 miRNAs might interact. Of these, 51% of them were oppositely expressed: one was upregulated as the other was downregulated. Due to their many-to-many interactions, many RNAs were repeatedly estimated. We found that 511 miRNAs and 59 circRNAs were downregulated. The miRNA-circRNA network based on potential relationships indicated that some circRNAs and miRNAs had a star-like distribution, suggesting potential crucial network positions (Figure 3).

A series of miRNA-circRNA interactions was obtained from starBase [46]. Based on the collected miRNA-circRNA interactions and the deregulated RNA molecules, we collected several abnormally expressed miRNAs, including 11 downregulated miRNAs (miR-124-3p, miR-129-5p, miR-135a-5p, miR-153-3p, miR-204-5p, miR-208a-3p, miR-211-5p, miR-218-5p, miR-488-3p, miR-490-3p, and miR-504-5p) and 1 upregulated miRNA (miR-373-3p). Most of the deregulated miRNAs with potential circRNA interactions were downregulated. Several circRNAs were detected in male patients with esophageal squamous cancer, mainly including circ-AGTPBP1, circ-ANKRD17, circ-GMPS, circ-PAPD4, and circ-SP140L. Interestingly, except for miR-373-3p:circ-SP140L (both upregulated), other miRNA:circRNA pairs had opposing deregulated expressions: in the cancer samples, miRNAs were downregulated and their paired circRNAs were upregulated (Table 1). Among the 97 deregulated circRNAs identified in starBase, 21 have been suggested to be competing endogenous RNAs (ceRNAs) of miRNAs. The paired miRNAs of these deregulated circRNAs were screened, focusing on the deregulated miRNAs without statistical difference (Table S2). A total of 19 pairs of potential circRNA:miRNA interactions were detected, and 14 of them (73.68%) were found to have opposing expression. Each member of these pairs might be involved in repeated circRNA and miRNA. For example, circ-SP140L was found to interact with 7 different miRNAs derived from several miRNA gene families.

We also found that target gene of miRNA may generate circRNA as ceRNA of the miRNA. For example, circ-GMPS and miR-217 might interact, and GMPS was also a potential target of miR-217 according to TargetScan [50].

### 3.4. isomiR-circRNA Interaction at the isomiR Levels

**Figure 4:**
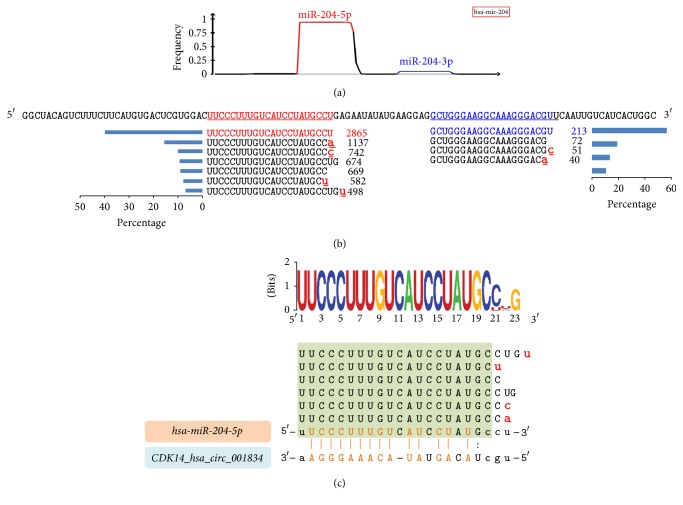
An example of isomiR expression and interaction with circRNA. (a-b) Hsa-mir-204 has two mature products, but miR-204-5p is the dominant miRNA generated. From each miRNA locus, multiple isomiR can be detected with various expression patterns and sequences. However, some of these isomiRs are more dominant and others are rare. (c) Sequence distribution patterns and interactions with circRNA. Multiple isomiRs may interact with the same circRNA, as long as the sequence in the binding region is homologous.

Based on the miRNA-circRNA pairs identified, we further investigated potential isomiR-circRNA interaction at the isomiR levels. For the dominantly expressed miRNA gene, both miR-#-5p and miR-#-3p might generate multiple isomiRs with various expressions (Figure 4(a)). Due to the expression characteristics of isomiRs, most isomiRs sequences are similar to the canonical miRNA sequence (Figure 4(b)), despite differences in expression and involvement with additional nontemplate nucleotides. Based on the potential binding characteristics of miRNA and circRNA, all of the relevant isomiRs from a given miRNA locus can also bind to the circRNA (Figure 4(c)). Thus, at the isomiR level, it is possible to detect many isomiR:circRNA pairs based on close sequence relationships. In order to survey these interactions, multiple isomiRs could first be clustered based on miRNA loci or different miRNA loci (some homologous miRNA loci generate similar isomiR sequences) (Figure 1(b)). Clustered specific miRNA sequences with enriched expressions and sequences could be used to predict miRNA-circRNA interactions. Thus, many types of small RNAs may interact with a specific circRNA, and these relationships are complex.

## 4. Discussion

Bioinformatic analysis indicates that 276 miRNAs bind to circRNAs according to starBase [46], and 2,603 mature miRNAs have been reported and validated according to miRBase (version 21.0) [42]. Many miRNA gene families are involved in these interactions; this may be because homologous miRNAs always have higher similarity. Only 10.60% of all identified miRNAs potentially interact with circRNAs. Most miRNAs are not involved in miRNA-circRNA interactions, while miRNA-mRNA interactions are quite popular. miRNA-circRNA interactions are not specific, and many small RNAs may interact with different molecules. These interactions may be important to regulatory processes, particularly those regulated by the small RNA. Some circRNAs that have been identified as miRNA sponges binding miRNA and reducing miRNA activity include ciRS-7/CDR1 [30, 34], circ-Sry [34], circ-ITCH [51, 52], circ-Foxo3 [51], circ-HRCR [53], circ-HIPK3 [54], and hsa_circ_001569 [33]. The ability of circRNA to act as miRNA sponges may also be important for the regulatory function of small RNAs. Herein, we aim to understand the two noncoding RNAs expression profiles and their interactions in male patients with esophagus cancer, focusing on the interactions at the isomiR level.

Based on sequence data, we collected several miRNA-circRNA pairs. Based on widespread miRNA gene families and gene clusters, many miRNA sequences are highly similar any may thus have similar expression patterns and functional relationships. Even though few miRNA-circRNA interactions were predicted when compared to the large numbers of RNA species, the details of the corresponding relationships vary substantially. However, similar to miRNA-mRNA and miRNA-miRNA interactions, the actual functional binding of the two ncRNAs* in vitro* remains unclear. Indeed, competitive relationships may also exist between multiple isomiRs. Of these isomiRs, many are repeatedly estimated due to high sequence similarity. miRNA-circRNA pairs are not as common as miRNA-mRNA pairs, but the role of potential miRNA sponges should be not ignored. For example, ciRS-7 abrogates miR-7 [55], circRNA_010567 suppress miR-141 [56], and circRNA_100290 may act as a sponge for the miR-29 gene family [57].

The miRNA-circRNA network that we constructed demonstrates the complex interaction patterns between the two ncRNAs (Figure 2). This network would increase in complexity complex if we included the detailed isomiRs. A given miRNA locus may generate multiple isomiRs with difference sequence and expression patterns. These isomiRs mainly differ at the 3′ ends. Thus, these isomiRs may potentially interact with circRNAs as well as their canonical miRNA sequence and isomiRs with high sequence similarity and similar expression relationships that are similar to homologous miRNAs [29]. Therefore, at the isomiR level, more complex interactions with circRNAs are possible, and miRNA sponges may absorb many types of small RNAs with similar sequences. The interesting phenomenon seems to enlarge interaction profiles as well as current pattern: a circRNA may be miRNA sponge for homologous miRNAs in a miRNA gene family, while multiple isomiRs can be considered a special larger gene family with high sequence similarity. At the isomiR level, the isomiR-circRNA interaction may be quite common and the network may be quite complex. Indeed, except for the interaction between isomiRs and circRNAs, interactions between miRNAs or isomiRs are also quite common, such as coordinated or restricted interactions, and these horizontal and vertical interactions increase RNA cross-talk. Moreover, based on the deregulated miRNAs and circRNAs we identified, the paired circRNAs may be derived from target gene of miRNA, although this seems rare compared to the number of miRNA-cirRNA interactions. CircRNA may suppress miRNA to increase the expression level of the miRNA target [34], simultaneously generating ceRNAs to control miRNAs to ensure gene expression.

In conclusion, based on the small RNA and circRNA expression profiles in male patients with esophagus cancer, some deregulated RNA species may show potential functional relationship, and their expression patterns are always opposed. Although fewer miRNAs are predicted to interact with circRNAs, the interaction between two ncRNAs is more common if detailed analysis is performed at the isomiR level. Similar to homologous miRNAs, isomiRs increase analysis and cross-talk complexity.

## Figures and Tables

**Table 1:** Selected deregulated miRNAs and their interacted deregulated circRNAs.

miRNA:circRNA	Deregulation pattern of miRNA:circRNA
miR-490-3p:circ-AGTPBP1	Down:Up
miR-490-3p:circ-ANKRD17	Down:Up
miR-124-3p:circ-ANKRD17	Down:Up
miR-490-3p:circ-GMPS	Down:Up
miR-124-3p:circ-PAPD4	Down:Up
miR-124-3p:circ-SP140L	Down:Up
miR-373-3p:circ-SP140L	Up:Up

*Note*: the miRNA-circRNA pairs are obtained based on deregulated miRNAs, and the circRNAs are named according to their genes.

## Supplementary Materials

Supplementary Materials Figure S1: clustering analysis showed expression distribution of circRNAs across different samples. Figure S2: Venn's distributions of expressed circRNAs between paired normal and tumor samples. Figure S3: clustering analysis and volcano distribution of miRNAs. Table S1: patients characteristics of 5 male patients with esophageal squamous cancer. Table S2: selected deregulated circRNAs and their interacted deregulated miRNAs.
