# Supplementary material for: Profiling and Bioinformatic Analyses Indicate Differential circRNA and miRNA/isomiR Expression and Interactions
Source: Biomed Res Int. 2018 Feb 25;2018:8518563. doi: 10.1155/2018/8518563 (PMC5845524; doi:10.1155/2018/8518563)
Supplement: Supplementary Materials — Figure S1: clustering analysis showed expression distribution of circRNAs across different samples. Figure S2: Venn's distributions of expressed circRNAs between paired normal and tumor samples. Figure S3: clustering analysis and volcano distribution of miRNAs. Table S1: patients characteristics of 5 male patients with esophageal squamous cancer. Table S2: selected deregulated circRNAs and their interacted deregulated miRNAs. [file 8518563.f1.docx]

**Supplementary Figures and Tables**


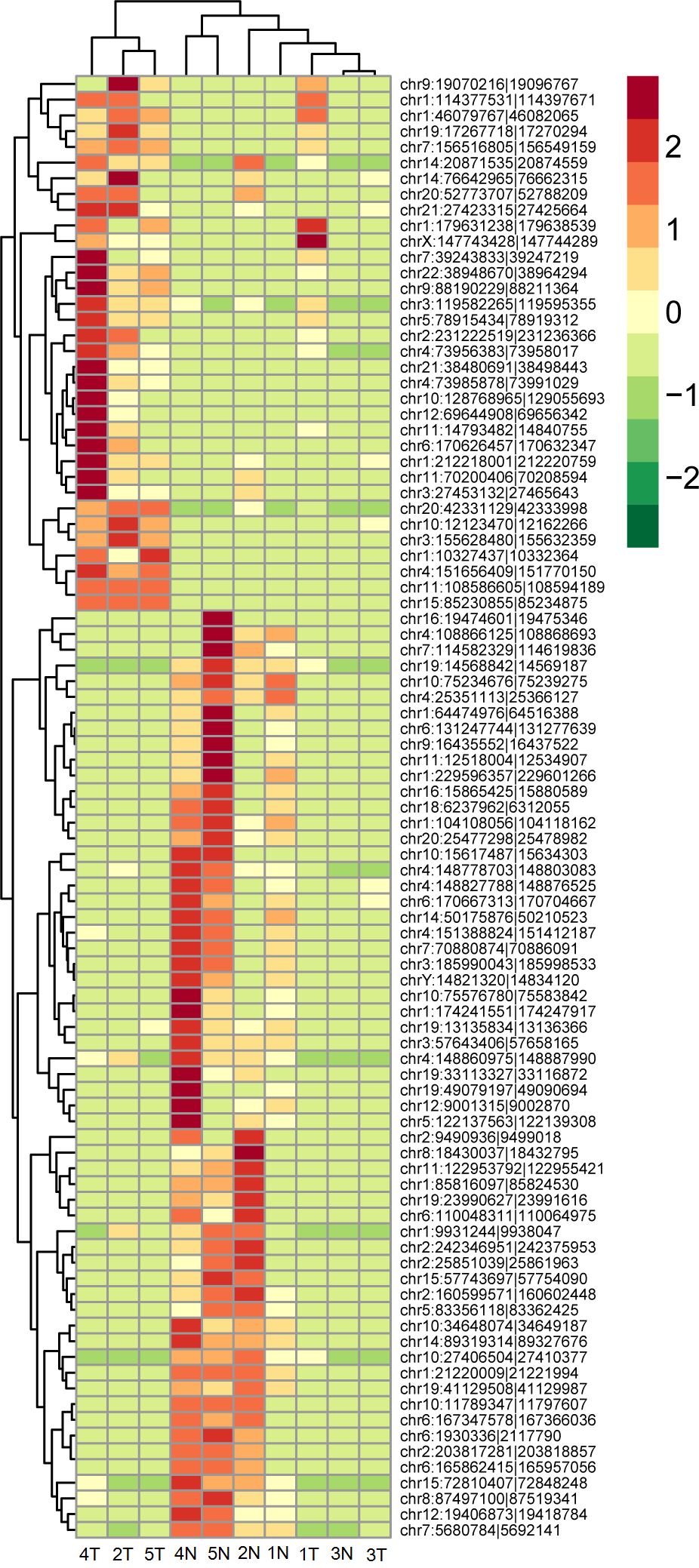


**Figure S1: Clustering analysis showed expression distribution of circRNAs across different samples.**

Note: The names of the circRNA are mainly their locations on chromosomes.


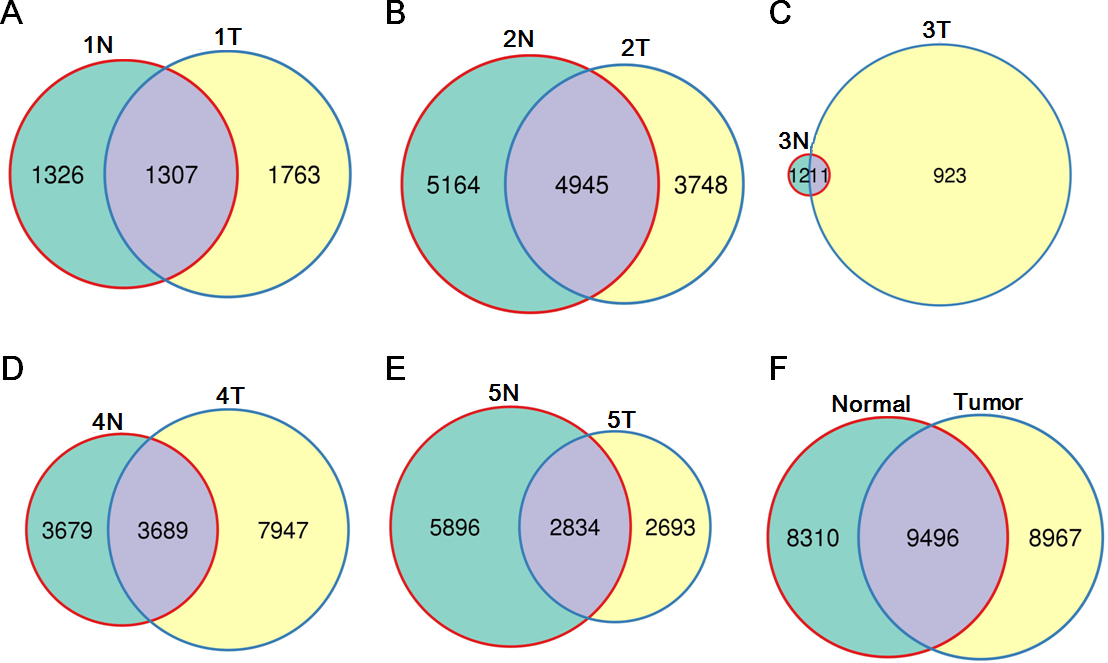


**Figure S2: Venn's distributions of expressed circRNAs between paired normal and tumor samples.**

A-E. Expressed circRNAs between paired normal and tumor samples; F. expressed circRNAs between all the pooled normal and tumor samples. The third samples were removed from the further analysis because fewer circRNAs were detected.


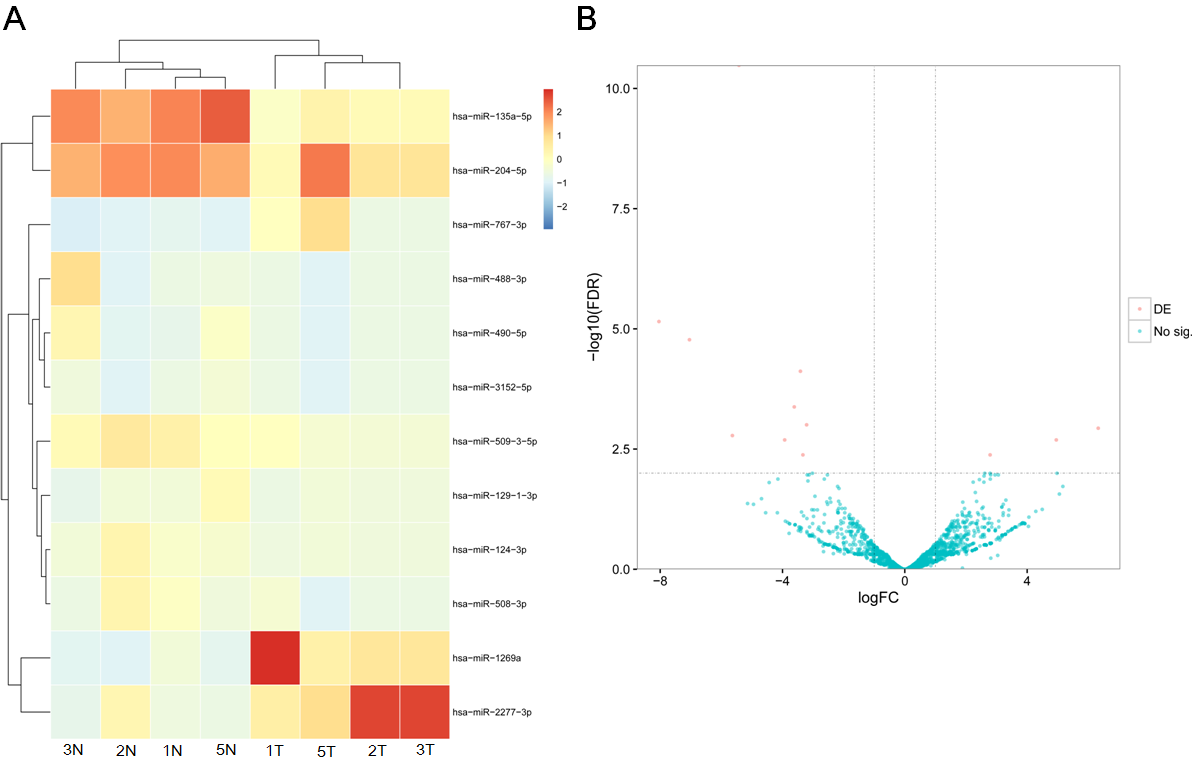


**Figure S3: Clustering analysis and** **volcano distribution of miRNAs.**

A. Clustering analysis of some miRNAs across different samples; B. volcano distribution of miRNAs.

Note: miRNAs in a specific sample (No. 4) were not involved in the relevant analysis because of outlier with other samples.

**Table S1: Patients characteristics of 5 male patients with esophageal squamous cancer.**

| **Age** | **Virus infection** | **History of smoking and drinking** | **Pathological condition** | **Lymph gland** |
| --- | --- | --- | --- | --- |
| 67 | no | Smoking for 30 years | Moderate differentiation | Reactive hyperplasia of lymph node |
| 60 | no | no | Medium-high differentiation | No metastasis |
| 67 | no | Intermittently, small amount of alcohol and smoking | Poor differentiation | Reactive proliferation |
| 63 | no | Intermittently, small amount of alcohol and smoking | Moderate or poor differentiation | Cancerometastasis |
| 64 | no | no | Moderate differentiation | Cancerometastasis |

**Table S2: Selected** **deregulated circRNAs and their interacted deregulated miRNAs.**

| **circRNA:miRNA** | **Deregulation pattern of circRNA:miRNA** |
| --- | --- |
| circ-AGTPBP1**:**miR-490-3p | Up**:**Down |
| circ-ANKRD17**:**miR-490-3p | Up**:**Down |
| circ-ANKRD17**:**miR-3118^#^ | Up**:**Down |
| circ-ANKRD17**:**miR-124-3p | Up**:**Down |
| circ-ARIH1**:**miR-26a-5p^#^ | Down**:**Down |
| circ-FAM120B**:**miR-133a-3p^#^ | Up**:**Down |
| circ-FAM120B**:**miR-133b^#^ | Up**:**Down |
| circ-GMPS**:**miR-217^#^ | Up**:**Down |
| circ-GMPS**:**miR-490-3p | Up**:**Down |
| circ-LRBA**:**miR-3118^#^ | Down**:**Down |
| circ-PAPD4**:**miR-124-3p | Up**:**Down |
| circ-SEC11A**:**miR-216b-5p^#^ | Up**:**Down |
| circ-SP140L**:**miR-520c-3p^#^ | Up**:**Up |
| circ-SP140L**:**miR-520d-3p^#^ | Up**:**Up |
| circ-SP140L**:**miR-373-3p | Up**:**Up |
| circ-SP140L**:**miR-216b-5p^#^ | Up**:**Down |
| circ-SP140L**:**miR-124-3p | Up**:**Down |
| circ-SP140L**:**miR-302d-3p^#^ | Up**:**Down |
| circ-SP140L**:**miR-302b-3p^#^ | Up**:**Down |

Note: the miRNA-circRNA pairs are obtained based on deregulated circRNAs.

^#^ shows the miRNA is found biological difference (log_2_FC > 2 or log_2_FC < -2) but no significant difference is detected (FDR > 0.05).
